# Impfung gegen SARS-CoV-2 bei Krebspatienten

**DOI:** 10.1007/s00761-021-00972-1

**Published:** 2021-05-17

**Authors:** Marie von Lilienfeld-Toal, Christina Rieger, Nicola Giesen, Bernhard Wörmann

**Affiliations:** 1 Schwerpunkt Infektionen in der Hämatologie und Onkologie, Klinik für Innere Medizin II, Abteilung für Hämatologie und Internistische Onkologie, Universitätsklinikum Jena, Am Klinikum 1, 07747 Jena, Deutschland; 2 Hämato-Onkologie Germering, Schwerpunktpraxis des Tumorzentrums München, Germering, Deutschland; 3 Abteilung für Hämatologie und Onkologie, Innere Medizin V, Universitätsklinikum Heidelberg, Heidelberg, Deutschland; 4 Medizinische Klinik mit Schwerpunkt Hämatologie, Onkologie und Tumorimmunologie, Charité – Universitätsmedizin Berlin, Berlin, Deutschland

**Keywords:** Immunsuppression, COVID-19, mRNA Impfstoff, Vektor-Impfstoff, Prävention, Immunosuppression, COVID-19, mRNA vaccine, vector-vaccine, prevention

## Abstract

Patient*innen mit Krebserkrankungen haben ein erhöhtes Risiko, schwere Verläufe einer COVID-19-Erkrankung zu erleiden. Spezifische Schutzmaßnahmen inkl. der COVID-19-Impfungen sind daher hier von besonderer Bedeutung. Eine durch Therapie und Grunderkrankung oftmals bedingte Abwehrschwäche kann jedoch eine Herausforderung für Impfstrategien bei diesen Patient*innen darstellen. Aktuell sind in Deutschland vier COVID-19-Impfstoffe zugelassen, zwei mRNA- und zwei vektorbasierte Impfstoffe, die alle eine sehr gute Wirksamkeit gegen schwere Krankheitsverläufe zeigen. Hinsichtlich des Wirkmechanismus ist allen vier Impfstoffen die Induktion einer Produktion virusspezifischer Proteine in menschlichen Zellen gemeinsam mit nachfolgender Aktivierung einer adaptiven Immunantwort. Für Krebspatient*innen und medizinisches Personal wird die Impfung priorisiert empfohlen. Ein optimaler Zeitpunkt für die Impfung bei Neuerkrankten scheint vor Beginn einer Tumortherapie zu sein. Aufgrund des erhöhten Risikos von Krebspatient*innen für schwere Verläufe von COVID-19 wird die Impfung jedoch auch unter laufender Tumortherapie empfohlen. Die Impfantwort ist hier allerdings möglicherweise reduziert. In den besonderen Konstellationen einer vorherigen Stammzelltransplantation oder einer B‑Zell-depletierenden Therapie wird nach Möglichkeit ein mehrmonatiger Abstand zwischen Therapie und Impfung empfohlen, da hier ansonsten mit einer deutlich reduzierten Impfantwort gerechnet werden muss. Wenn sich erste Hinweise auf nur eine geringe Serokonversion bei Krebspatient*innen nach einmaliger Impfung bestätigen, können zukünftige Empfehlungen in Richtung mehrfacher Impfungen bei diesen Patient*innen gehen.

## Abstract (en)

Patients with cancer are at an increased risk to suffer severe coronavirus disease 2019 (COVID-19). Therefore, specific preventative measures including COVID-19 vaccines are especially important. Both anticancer therapies and the underlying malignancy itself can lead to significant immunosuppression posing a particular challenge for vaccination strategies in these patients. At the moment, four COVID-19 vaccines are European Medicines Agency (EMA) approved in Germany: two mRNA and two viral vector-based vaccines. All four vaccines show excellent protection against severe COVID-19. Their mechanism of action relies on the induction of the production of virus-specific proteins by human cells and the following activation of a specific adaptive immune response. Vaccination against COVID-19 has been prioritized for cancer patients and medical personnel in Germany. Regarding timing of vaccination, vaccination prior to initiation of anticancer therapy seems ideal in newly diagnosed disease. However, due to the significant risk of severe COVID-19 in cancer patients, vaccination is also strongly recommended for patients already undergoing anticancer therapy. In these patients, immune response might be reduced. In two particular patient cohorts, namely stem cell transplant recipients and patients treated with B‑cell depleting agents, an interval of several months following therapy is recommended because otherwise the response to vaccination will most likely be severely reduced. Preliminary data suggest only low rates of seroconversion following a single shot of vaccine in cancer patients. Therefore, on the long run, repeat vaccination regimens might be preferable in cancer patients.

## Hintergrund zu COVID-19 und Krebs

Krebspatient*innen haben als Kohorte ein höheres Risiko eines schweren Verlaufs einer COVID-19-Erkrankung als die Allgemeinbevölkerung. Dies liegt vor allem daran, dass Krebspatient*innen oft älter sind und häufiger Komorbiditäten aufweisen. Innerhalb der Gruppe der Krebspatient*innen sind diejenigen mit einer aktiven, kürzlich diagnostizierten Erkrankung oder mit einer hämatologischen Neoplasie besonders gefährdet [12]. Insofern ist es folgerichtig, insbesondere für Krebspatient*innen den Schwerpunkt auf die Prävention von COVID-19 durch Allgemeinmaßnahmen und zunehmend auch durch die Impfung zu legen.

## Funktionsweise der COVID-19-Impfstoffe

Zahlreiche, sehr unterschiedliche Ansätze werden derzeit zur raschen Produktion wirksamer Schutzimpfungen verfolgt [1, 10, 18, 19]. Die zugelassenen Impfstoffe zeichnen sich alle dadurch aus, dass virale mRNA in menschlichen Zellen zu virustypischen Proteinen (in der Regel Spike-Proteine) umgeschrieben und sezerniert werden. Diese Proteine wiederum werden durch antigenpräsentierende Zellen aufgenommen und führen in Lymphknoten zu einer adaptiven Immunantwort mit der Bildung neutralisierender Antikörper sowie spezifischer T‑Zellen. Es gibt zwei verschiedene Wege, die Virus-mRNA in die menschlichen Zellen zu bringen: die so genannten mRNA-Impfstoffe enthalten mit Trägermolekülen umhüllte mRNA. Diese Trägermoleküle (häufig Fettmoleküle beziehungsweise Nanopartikel) stabilisieren die sonst sehr leicht zerfallende RNA, sodass sie von den menschlichen Zellen aufgenommen und umgeschrieben werden kann. Im Gegensatz dazu benutzen die Vektorimpfstoffe einen viralen Vektor, in der Regel rekombinationsdefiziente Adenoviren, der DNA enthält. Vektor und Inhalt werden in die menschliche Zelle aufgenommen, die DNA wird im Zellkern zu RNA umgeschrieben, und der weitere Werdegang ist im Wesentlichen wie bei den mRNA-Impfstoffen.

**Figure 1:**
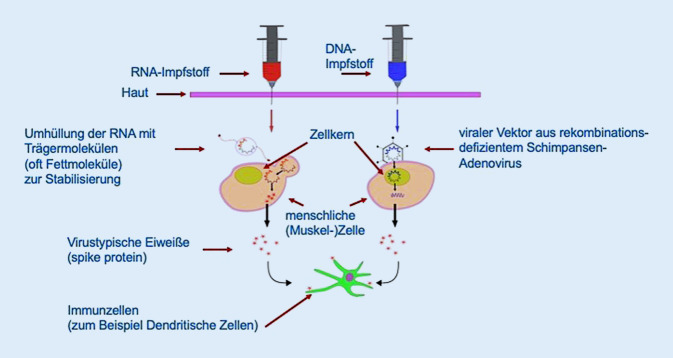

Da die viralen Vektoren aus rekombinationsdefizienten Viren hergestellt werden, die einen Enzymdefekt haben, der ihnen ein Wachstum nur in entsprechend substituiertem Medium erlaubt, gelten diese Impfstoffe als Totimpfstoffe. Auch die mRNA-Impfstoffe sind Totimpfstoffe, da sie keinerlei lebendes Material enthalten. Beide Impfstofftypen sind nicht als Gentherapie anzusehen, da die mRNA-Impfstoffe vollkommen im Zytoplasma verbleiben und nicht mit dem menschlichen Genom in Berührung kommen. Die DNA der Vektorimpfstoffe wird zwar im Zellkern umgeschrieben, aber nicht in das menschliche Genom integriert und kann auch nicht weitergegeben werden. Somit erfolgt auch hier keine Gentherapie (Abb. 1).

## Wirksamkeit der zugelassenen COVID-19-Impfstoffe

**Figure 2:**
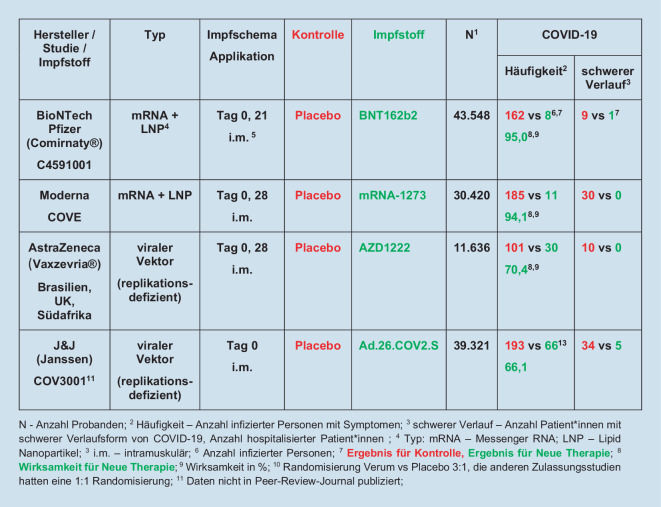

Bisher sind 4 Impfstoffe in der EU zugelassen. Die Daten dieser Zulassungsstudien sind publiziert und/oder in den Zulassungsunterlagen hinterlegt [8]. Charakteristika der Impfstoffe und Ergebnisse zur Wirksamkeit sind in Abb. 2 zusammengefasst.

Die häufigsten Nebenwirkungen sind eine Lokalreaktion an der Injektionsstelle, die vor allem mit Schmerzen einhergeht. Systemische Nebenwirkungen wie Fatigue, Kopfschmerzen oder Fieber treten ebenfalls bei etwa der Hälfte der Geimpften auf. Interessanterweise erleiden vor allem jüngere und weibliche Menschen Nebenwirkungen. Nach Verabreichung der mRNA-Impfstoffe sind die Nebenwirkungen nach der zweiten Injektion ausgeprägter als nach der ersten, während dies nach Verabreichung der AstraZeneca-Vektorvakzine umgekehrt der Fall ist. Sehr selten treten nach den mRNA-Impfstoffen anaphylaktische Reaktionen auf. Diese traten bei der Mehrheit der Betroffenen innerhalb von 15 min nach der Injektion auf. Deshalb sollte nach der Verabreichung des Impfstoffs immer eine angemessene medizinische Versorgung und Überwachung bereitstehen. Nach der Impfung wird eine engmaschige Beobachtung von mindestens 15 min empfohlen. Eine zweite Dosis des Impfstoffs sollte nicht an Personen verabreicht werden, bei denen eine Anaphylaxie nach der ersten Dosis aufgetreten ist.

Im zeitlichen Zusammenhang mit der Applikation der Vektorvakzinen zur COVID-19-Schutzimpfung sind Sinus- und Hirnvenenthrombosen (CVST) mit systemischen Gerinnungsstörungen und z. T. schweren klinischen, auch tödlichen Verläufen beobachtet worden [3, 5, 14, 16]. Beispielsweise wurde sehr selten nach einer Impfung mit Vaxzevria von AstraZeneca eine Kombination von Thrombose und Thrombozytopenie, in einigen Fällen einhergehend mit Blutungen, beobachtet. Dies schließt schwere Fälle ein, die sich als venöse Thrombose präsentierten, einschließlich des Auftretens in ungewöhnlichen Bereichen, wie zum Beispiel *zerebrale Sinusvenenthrombose, Mesenterialvenenthrombose sowie arterielle Thrombose*, bei gleichzeitiger Thrombozytopenie. Die meisten dieser Fälle traten innerhalb der ersten sieben bis vierzehn Tage nach der Impfung und bei Frauen unter 55 Jahren auf, was möglicherweise auf die verstärkte Anwendung des Impfstoffs in dieser Bevölkerungsgruppe zurückzuführen ist. Einige Fälle hatten einen tödlichen Ausgang [5]. Weltweit wurde unterschiedlich reagiert. Diese schweren Gerinnungsstörungen sind sehr selten, die genaue Häufigkeit ist unklar. Frauen <55 Jahren sind häufiger betroffen. Es gibt bisher keine Hinweise, dass Patient*innen mit vorbestehenden, hereditären oder erworbenen Gerinnungsstörungen ein erhöhtes Risiko für diese Komplikationen haben. Derzeit werden für diese Komplikation die Begriffe „vaccine-induced immune thrombotic thrombocytopenia“ (VITT) oder vakzineinduzierte prothrombotische Immunthrombozytopenie (VIPIT) gebraucht. Ein möglicher Pathomechanismus ist die Induktion einer Antikörperbildung gegen Thrombozytenantigene im Rahmen der vakzineinduzierten Immunstimulation. Diese Antikörper induzieren eine Thrombozytenaktivierung über den Fc-Rezeptor in Analogie zur heparininduzierten Thrombozytopenie (HIT). Die Gesellschaft für Thrombose- und Hämostaseforschung (GTH) und andere Fachgesellschaften haben Empfehlungen für die Diagnostik bei vorliegendem Verdacht und für den Umgang mit diesen Gerinnungsstörungen publiziert [9]. Gerinnungsstörungen in der Vorgeschichte einschl. Thrombosen sind keine Kontraindikation gegen eine Impfung mit der AstraZeneca-Vakzine. Während seitens der EMA und der WHO aktuell keine Einschränkungen des Einsatzes der AstraZeneca-Vakzine formuliert wurden, empfiehlt in Deutschland die STIKO den Einsatz der AstraZeneca-Vakzine nun primär für Personen ≥60 Jahren, wobei nach entsprechender Aufklärung und individueller Risiko-Nutzen-Abwägung weiterhin auch der Einsatz bei jüngeren Patienten möglich bleibt.

Neben den in Abb. 2 zusammengefassten Daten der Zulassungsstudien sind auch Erfahrungen aus der Versorgung (Real World Data) relevant. Dabei zeigte sich im schottischen Register mit 5,4 Mio. Personen bereits nach der ersten Schutzimpfung mit der AstraZeneca-Vakzine ein Rückgang in der COVID-19-bezogenen Hospitalisationsrate um 95 % [17].

Inzwischen wurden in den Medien auch Ergebnisse einer Interimsanalyse der erweiterten Zulassungsstudie bei Personen in den USA, Chile und Peru berichtet. Sie bestätigen die Daten der Zulassung, sind aber bisher nicht voll in einem Peer-Review-Journal publiziert.

Weltweit gibt es eine Reihe weiterer Impfstoffe. Dazu gehören u. a. der Impfstoff der Fa. Sinovac Biotech, Sputnik V und der Impfstoff der Fa. CureVac [7]. Sie sind bisher nicht für die EU zugelassen. Bisher ist nicht vollständig geklärt, inwieweit die zugelassenen Schutzimpfungen auch gegen mutierte Viren („variants of concern“) wirksam sind. Die bisher vorliegenden Daten zeigen, dass die für die EU zugelassenen Impfstoffe auch gegenüber der aktuell (Stand 04/2021) in Deutschland dominanten Variante B.1.1.7. einen wirksamen Schutz bieten, was aber möglicherweise nicht auf alle existierenden Varianten zutrifft [4, 20].

## Empfehlungen zur Schutzimpfung gegen COVID-19 bei Krebspatient*innen

Krebspatient*innen und Menschen mit Immunsuppression aufgrund hämatologischer Erkrankungen sowie das betreuende medizinische Personal werden in Deutschland wegen des erhöhten Risikos für einen schweren Verlauf in eine hohe Prioritätsstufe für die Impfung eingeordnet. Auch bis zu 2 enge Kontaktpersonen aus dem Angehörigenkreis pflegebedürftiger Krebspatient*innen haben eine erhöhte Priorität für die Impfung. Es liegen trotz der weltweit millionenfachen Impfungen gegen COVID-19 bisher für die hier betrachtete Gruppe der Patient*innen mit Krebserkrankungen nur zahlenmäßig begrenzte und vorläufige Daten vor, sodass Entscheidungen und Empfehlungen teilweise auf Analogien zu Impfungen gegen Influenza bei dieser Patientengruppe zurückgreifen.

### Zeitpunkt der Impfung

Der Schutz einer COVID-19-Impfung kann bei immunsupprimierten Patient*innen geringer sein, erste Daten deuten auf eine erniedrigte Antikörperbildung nach einer einmaligen Impfung hin [8]. Umfassende Daten liegen dazu nicht vor, auch nicht zu einer schädlichen Wirkung der Impfstoffe. In der praktischen Versorgung empfehlen wir, folgende Aspekte zu berücksichtigen:

#### Geplante Operation oder Bestrahlung

- Wenn immer möglich, sollte die Schutzimpfung vor Beginn einer onkologischen Therapie erfolgen. Das betrifft auch chirurgische Eingriffe oder eine Bestrahlung.

#### Chemotherapie

- Wenn immer möglich, sollte die Schutzimpfung vor Beginn einer systemischen Therapie erfolgen.
- Während einer laufenden zyklischen Chemotherapie sollte versucht werden, einen zeitlichen Abstand zur systemischen Therapie zu erreichen, um das Risiko überlappender Nebenwirkungen zu vermindern. Daten prospektiver Studien zum optimalen Zeitpunkt der COVID-19-Schutzimpfung während einer systemischen Therapie liegen nicht vor. Ein Aussetzen oder Verschieben der Chemotherapie wird nicht routinemäßig empfohlen. Daten zur Influenza-Schutzimpfung mit Booster-Impfungen zeigen, dass der Abstand zur Chemotherapie hinsichtlich der Impfwirkung keine wesentliche Rolle spielt [11].

#### Immunsuppressive Therapie

- Auch unter einer fortlaufenden immunsuppressiven Therapie gibt es keinen optimalen Zeitpunkt für die Schutzimpfung. Ein Aussetzen der Therapie wird nicht empfohlen. Analog zur Chemotherapie ist in der Regel bei einer Impfung mit Booster von einer guten Impfantwort auszugehen.
- Eine Ausnahme bildet die B‑Zell-depletierende Therapie mit Anti-CD-20-Antikörpern, bispezifischen Antikörpern, Antikörperkonjugaten, Anti-CD19-CAR-T-Zellen oder BTKi (Bruton-Tyrosinkinase-Inhibitoren)/bcl2-Inhibitoren („B-cell lymphoma 2“). Hier ist zu erwarten, dass – in Analogie zur Influenza-Schutzimpfung – die Impfantwort trotz Booster wahrscheinlich deutlich reduziert ist. Hierfür sprechen Daten einer deutlich erniedrigten Serokonversion bei diesen Patienten [6]. Daher kann ein zeitlicher Abstand von mindestens 3 Monaten oder sogar länger zur letzten Therapie erwogen werden. In anderen Empfehlungen wird sogar ein Abstand von bis zu 6 Monaten empfohlen. Allerdings beruhen diese Empfehlungen auf der Annahme, dass möglicherweise kein wirksamer Schutz aufgebaut wird und nicht auf einer schädlichen Wirkung. Da der Schutz durch die COVID-19-Vakzine jedoch auch T‑Zell-vermittelt ist [13], stellt die B‑Zell-Depletion an sich keine absolute Kontraindikation für die COVID-19-Schutzimpfung dar, und es kann im Einzelfall sinnvoll sein, trotz fehlender B‑Zellen in einer Hochrisikokonstellation zu impfen. Aufgrund des verminderten Ansprechens sollten diese Patient*innen ausdrücklich auf das weitere Einhalten der Hygieneregeln hingewiesen werden.
- Auch Patient*innen vor oder nach einer allogenen Stammzelltransplantation bilden eine eigene Gruppe. Die COVID-19-assoziierte Mortalität ist nach einer allogenen Stammzelltransplantation möglicherweise erhöht [15]. Deshalb sollte, wenn immer möglich, die Schutzimpfung vor Beginn einer systemischen Therapie erfolgen. Zur Schutzimpfung nach einer allogenen Stammzelltransplantation liegen Empfehlungen der European Society for Blood and Marrow Transplantation vor (https://www.ebmt.org/covid-19-and-bmt). Diese beinhalten einen Abstand von mindestens 3 Monaten nach der Transplantation sowie eine Verzögerung der Schutzimpfung bei unkontrollierter Graft-versus-Host-Disease, nach B‑Zell-depletierender oder nach CAR-T-Zell-Therapie sowie nach Therapie mit ATG oder Alemtuzumab.

Bei der patientenindividuellen Entscheidungsfindung über die Durchführung einer COVID-19-Impfung gelten die Grundsätze des Shared Decision Making zwischen Arzt und Patient*in unter besonderer Berücksichtigung der individuellen Risikosituation.

Die bisher zugelassenen Impfstoffe werden intramuskulär appliziert. Eine subkutane Applikation kann die Wirksamkeit beeinträchtigen und wird daher nicht empfohlen. Bei Patient*innen mit klinischer manifester Blutungsneigung (Thrombozytopenie, Antikoagulation o. a.) wird eine ausreichend lange Kompression an der Injektionsstelle und gegebenenfalls das Anliegen eines Stauschlauchs für 2–3 min nach der Injektion empfohlen. Ein Aussetzen der Antikoagulation wird nicht empfohlen, bei ausgeprägter Thrombozytopenie kann die Gabe eines Thrombozytenkonzentrats erwogen werden.

Bei Patient*innen mit der Vorgeschichte einer anaphylaktischen Reaktion soll das Risiko einer schweren Nebenwirkung besonders sorgfältig gegenüber dem erwarteten Nutzen abgewogen werden. Grundsätzlich wird für diese Patienten ein längeres Nachbeobachtungsintervall nach der Impfung in der Impfpraxis/dem Impfzentrum empfohlen.

Die zuletzt beschriebenen thromboembolischen Ereignisse in Zusammenhang mit vektorbasierten Impfstoffen (VITT, VIPIT, s. oben) sind unabhängig von Tumorerkrankungen aufgetreten. Bisher liegen keine Hinweise vor, dass diese Komplikationen bei Patient*innen mit Krebserkrankungen häufiger vorkommen.

Mit Ausnahme der Vakzine von Johnson & Johnson ist für alle bisher in der EU zugelassenen Impfstoffe eine zweite Impfung vorgesehen. In den Zulassungsstudien erfolgte diese nach 21 bzw. 28 Tagen (Abb. 2). Aufgrund der aktuellen Engpasssituation in der Impfstoffverfügbarkeit wird das Intervall bis zur zweiten Impfung häufig verlängert, auf bis zu 12 Wochen.

Daten einer aktuellen vergleichenden Studie zu BNT162b2 (BioNTech/Pfizer) deuten darauf hin, dass die Serokonversion bei Krebspatient*innen nach der ersten Schutzimpfung deutlich geringer als bei Gesunden ausfällt mit diesen Raten: 40 % bei Patient*innen mit soliden Tumoren, 15 % bei hämatologischen Neoplasien, >90 % bei Gesunden. Nach der zweiten Impfung wurde bei Patient*innen mit soliden Tumoren eine Serokonversion von 95 % erreicht [8]. Niedrigere Serokonversionsraten nach einmaliger Impfung wurden auch bei Z. n. Organtransplantation beobachtet [2]. Diese Daten deuten darauf hin, dass bei Verwendung eines mRNA-Impfstoffs die zweite Schutzimpfung bei Krebspatient*innen zeitnah, d. h. nach 3 bzw. 4 Wochen, stattfinden sollte. Bei vektorbasierten Impfstoffen kann ein längeres Intervall sinnvoll sein.

Möglicherweise ist nach stattgehabter Impfung von ausgeprägt immunsupprimierten Patient*innen nach Regeneration eine spätere, erneute Schutzimpfung sinnvoll. Eine Empfehlung zu dieser Indikation wird erst nach Vorliegen weiterer Daten möglich sein. Offen ist auch, ob bei Patient*innen nach durchgemachter COVID-19-Erkrankung eine einmalige Impfung zur Boosterung entsprechend der aktuellen STIKO-Empfehlung ausreicht oder ob eher eine vollständige Schutzimpfung erforderlich ist.

### Wahl des Impfstoffs bei Krebspatient*innen

Die Wirksamkeit der beiden mRNA-basierten Impfstoffe ist etwa gleich hoch. Unterschiede finden sich vor allem in der Lagerung, der Applikation und im Nebenwirkungsspektrum. Die vektorbasierten Impfstoffe zeigen eine etwas geringere Wirksamkeit im Schutz vor Infektionen, siehe Abb. 2. Allerdings ist der Schutz vor schweren Verläufen von COVID-19 bei den zugelassenen Impfstoffen gleich hoch. Es gibt bisher auch keine Hinweise auf eine höhere Rate von Nebenwirkungen bei Krebspatient*innen nach der Schutzimpfung.

Entsprechend gibt es bisher keine Evidenz für Empfehlungen für oder gegen einen der zugelassenen Impfstoffe. Derzeit ist jeder Impfstoff besser als kein Impfstoff. Allerdings suggerieren die o. a. Daten, dass eine Einmal-Schutzimpfung bei Krebspatient*innen nicht ausreichend ist.

Langzeitergebnisse zur Dauer der Wirksamkeit der Impfung und zur Sicherheit liegen für keinen der Impfstoffe vor.

## Fazit für die Praxis

- Eine COVID-19-Schutzimpfung sollte routinemäßig allen Krebspatient*innen angeboten werden.
- Neben einer bekannten Anaphylaxie gibt es praktisch keine Kontraindikationen gegen die Impfung, wenn auch teilweise von einer reduzierten Immunantwort ausgegangen werden muss.
- Ungefährliche Nebenwirkungen wie lokale Reaktionen und Fatigue sind häufig und vor allem bei jüngeren Frauen zu erwarten, wobei die mRNA-Impfstoffe vor allem nach der 2. und der Vektorimpfstoff vor allem nach der 1. Injektion Nebenwirkungen hervorruft.
- Bei Krebspatient*innen sind Impfregime mit Doppelimpfung aufgrund des erwarteten besseren Ansprechens zu bevorzugen.
